# Research progress on the use of beta-blockers in the progression prevention, and treatment of retinopathy of prematurity

**DOI:** 10.3389/fped.2026.1953347

**Published:** 2026-09-22

**Authors:** Shuanghong Bai, Huiqing Sun

**Affiliations:** Department of Neonatology, Children’s Hospital Affiliated with Zhengzhou University, Henan, China

**Keywords:** beta-blockers, propranolol, retinopathy of prematurity (ROP), topical ocular administration, vascular endothelial growth factor

## Abstract

Retinopathy of prematurity (ROP) is a proliferative retinal vascular disorder unique to preterm infants with extremely low birth weight, ranking as the top cause of preventable childhood blindness. Retinal hypoxia, excessive secretion of vascular endothelial growth factor (VEGF) and dysregulation of insulin-like growth factor-1 (IGF-1) constitute the core pathogenic cascades of ROP. As competitive antagonists of *β*-adrenergic receptors (*β*-AR), beta-blockers suppress the HIF-1*α*/VEGF and PI3K/Akt/VEGF signaling axes to inhibit pathological retinal neovascularization, while exerting auxiliary anti-inflammatory, anti-oxidative stress and retinal neuroprotective effects. Derived from the mature clinical application of propranolol in infantile hemangioma (IH), preclinical and clinical trials of beta-blockers for the prevention of ROP progression and for ROP treatment have expanded rapidly over the past decade, with two administration routes available: oral systemic delivery and topical ocular micro-drops. Oral propranolol possesses solid anti-angiogenic preclinical evidence, yet its clinical promotion is restricted by severe systemic adverse reactions including bradycardia, hypoglycemia and apnea. The 0.2% propranolol eye micro-drops carry minimal systemic exposure risk and deliver comparable efficacy to oral formulations for Stage 2–3 ROP, but long-term visual and neurodevelopmental follow-up data remain absent. Critically, all existing clinical evidence is limited by small sample sizes, single-center designs and inconsistent dosing protocols; large-scale, multicenter, long-term follow-up Phase III randomized controlled trials are still lacking. At present, beta-blockers cannot replace laser photocoagulation or intravitreal anti-VEGF injection as the routine standard treatment for ROP. They are only regarded as experimental early intervention candidates pending further high-level clinical validation.

## 1 Introduction

ROP is an ischemic proliferative retinal disease predominantly affecting preterm infants, particularly those with gestational age < 32 weeks or birth weight < 1,500 g, who are at the highest risk; it imposes a heavy global visual disability burden on neonatal populations (1). The physiological arrest of retinal vascular development induced by prematurity, combined with postnatal hyperoxia exposure and blood oxygen fluctuation, triggers two-stage pathological progression: avascular retinal ischemia followed by hypoxia-driven pathological neovascularization, eventually leading to tractional retinal detachment and irreversible blindness in severe cases (2). Current first-line standard interventions for ROP include laser photocoagulation and intravitreal anti-VEGF monoclonal antibody injection (3, 4). Both therapies achieve definite curative effects for treatment-requiring ROP, yet they carry inherent limitations: laser treatment causes permanent retinal tissue damage, while intravitreal injection is an invasive operation with risks of endophthalmitis and repeated anesthesia demand (5). While the short-term efficacy of these established therapies is well documented, their long-term safety profiles—particularly regarding retinal structural damage from laser and systemic effects of VEGF suppression from intravitreal injections—are not without uncertainty. Moreover, neither approach enables early preventive intervention for mild ROP at Stage 1–2, generating an urgent clinical demand for safe, non-invasive early therapeutic strategies. Neither option can implement early preventive intervention for mild ROP at Stage 1–2, generating an urgent clinical demand for safe, non-invasive early therapeutic strategies. Beta-blockers, represented by non-selective propranolol, have been established as first-line systemic therapy for high-risk IH over the past 15 years (6). IH and ROP share identical core pathological driver: hypoxia-mediated HIF-1*α* overactivation and subsequent VEGF overexpression, which provides a translational theoretical basis for repurposing beta-blockers to treat ROP. A large body of oxygen-induced retinopathy (OIR) animal experiments have verified the anti-angiogenic, barrier-protective and neuroprotective properties of beta-blockers, and multiple small-scale clinical trials have explored oral and topical ocular delivery regimens. Nevertheless, after more than ten years of translational research, beta-blockers have not been incorporated into routine ROP clinical management. Conflicting trial efficacy outcomes, systemic toxic risks of oral formulations, insufficient long-term safety data, and mature competing standard therapies all form major translational barriers. This review aims to: (1) elaborate the differential regulatory mechanisms of *β*1/β2/*β*3-adrenergic receptor subtypes in retinal angiogenesis; (2) dissect multi-target signaling pathways of beta-blockers against ROP pathological injury; (3) critically appraise the quality, consistency and limitations of clinical evidence for oral vs. topical propranolol; (4) summarize core obstacles restricting clinical routine application; (5) propose future high-priority research directions to fill existing evidence gaps.

## 2 Pathogenesis of ROP

ROP originates from interrupted physiological retinal vascular maturation secondary to preterm birth, with combined congenital and postnatal risk factors jointly driving disease progression. Fetal retinal vasculogenesis initiates at gestational week 16 and completes at week 40 (7). Preterm infants delivered before 32 weeks present extensive avascular peripheral retina at birth (8). The pathological course is divided into two sequential phases:

### 2.1 Vascular occlusion (ischemic) phase

Prolonged hyperoxia exposure directly suppresses retinal VEGF transcription, inducing retinal arteriolar vasoconstriction, capillary occlusion and complete stagnation of physiological angiogenesis, forming permanent avascular retinal zones (2). Meanwhile, circulating insulin-like growth factor-1 (IGF-1) levels decline sharply. As an indispensable synergistic co-factor of VEGF for endothelial proliferation, IGF-1 deficiency further blocks compensatory retinal vascular regrowth.

### 2.2 Vascular proliferation phase

As retinal metabolic oxygen demand rises with postnatal maturation, the avascular retinal region enters relative hypoxia, activating HIF-1*α*. HIF-1*α* acts as a master transcription factor to upregulate VEGF, erythropoietin (EPO) and other pro-angiogenic mediators, stimulating uncontrolled pathological proliferation of vascular endothelial cells into the vitreous cavity (9). Concurrent complement system activation exacerbates lesion severity: activated complement fragments C3a and C5a recruit and polarize retinal microglia and macrophages, triggering massive release of pro-inflammatory cytokines and additional VEGF to amplify abnormal neovascularization (10, 11). Disorganized pathological new vessels are prone to plasma leakage and intraretinal hemorrhage, accompanied by progressive perivascular fibrosis. Contraction of fibrous scar tissue generates tractional forces on the retina, resulting in tractional retinal detachment and permanent visual loss in advanced ROP (12).

## 3 Treatment of infantile hemangiomas with beta-blockers

IH is a benign vascular tumor that commonly occurs in infancy. It grows rapidly during the first year of life; while most patients do not require treatment, approximately 12% of affected children require standardized and effective treatment due to factors such as the location and size of the hemangioma, as well as any complications it may cause. The efficacy of beta-blockers, particularly propranolol, in the treatment of IH is supported by clinical and evidence-based medical data. From a molecular mechanism perspective, beta-blockers exert their therapeutic effects through multiple pathways and targets, such as inhibiting angiogenesis, inducing endothelial cell apoptosis, and modulating the cell cycle and gene expression. Propranolol can induce apoptosis in hemangioma endothelial cells by activating intrinsic and extrinsic apoptotic pathways, modulating the p53-BAX signaling pathway, and inhibiting the AKT/mTOR pathway (13–15). At the same time, propranolol can inhibit the angiogenesis process in hemangiomas by regulating angiogenesis-related factors such as VEGF, HIF-1*α*, and MMPs (16). The primary mechanism by which propranolol treats infantile hemangiomas is its ability to significantly downregulate key angiogenesis signaling pathways, particularly the VEGF and HIF-1*α* pathways (17, 18). Under hypoxic conditions, HIF-1*α* is the primary transcription factor driving VEGF expression; propranolol has been shown to inhibit the stability and activity of HIF-1*α*, thereby reducing VEGF production at its source (19). This mechanism aligns closely with the core process of hypoxia-driven pathological angiogenesis in ROP. Multi-system evaluations and meta-analyses have confirmed that oral propranolol is the first-line treatment for infantile hemangiomas, with an efficacy rate far higher than that of placebo or other conventional therapies (20, 21). Oral propranolol is currently the first-line treatment for infantile hemangiomas, and numerous clinical studies have confirmed its high efficacy and safety (22). For superficial, localized infantile hemangiomas, topical application of beta-blockers (such as timolol, betaxolol, etc.) is also an effective treatment option (20). Given propranolol's established anti-angiogenic effects in the treatment of IH (23)and its potential link to the pathophysiology of ROP, researchers have begun to systematically evaluate the potential of beta-blockers as a new strategy for the prevention or treatment of ROP.

## 4 Beta-blockers: mechanism of action against ROP

### 4.1 Distribution and functional divergence of *β*-adrenergic receptor subtypes

*β*-adrenergic receptors (*β*-AR) belong to the G protein-coupled receptor superfamily, consisting of three distinct subtypes with divergent tissue distribution (24), downstream G-protein coupling and biological functions, which underpin the complex regulatory effects of beta-blockers on retinal angiogenesis. The classic signaling pathway of *β*-ARs is mediated by their coupling to the stimulatory G protein (Gs) within the heterotrimeric G protein complex. When a receptor is activated by an agonist (such as epinephrine), Gs protein is activated, which in turn activates the downstream effector, adenylyl cyclase (AC). AC catalyzes the conversion of ATP into the second messenger cyclic adenosine monophosphate (cAMP). Elevated cAMP levels exert their biological effects primarily by activating protein kinase A (PKA) (25). *β*1-AR, primarily expressed in cardiac myocytes, with low-level expression in retinal endothelial cells. It couples with stimulatory Gs to activate the AC-cAMP-PKA cascade and promote VEGF production, yet its independent contribution to ROP pathogenesis remains poorly characterized with limited targeted experimental evidence. *β*2-AR, the dominant therapeutic target for ROP intervention, highly expressed in retinal Müller glial cells and capillary endothelial cells (26, 27). *β*2-AR activation robustly upregulates VEGF secretion (28), accelerates endothelial migration and proliferation, and disrupts tight junction integrity of the blood-retinal barrier (BRB) (29). Genetic polymorphism studies confirm ADRB2 gene variants correlate with elevated ROP incidence risk, verifying its central pathogenic role (30). *β*3-AR, predominantly localized to retinal capillary endothelial cells (24), with unique signal transduction characteristics differing from *β*1/β2-AR. *β*3-AR preferentially couples with inhibitory G protein (Gi) to reduce intracellular cAMP concentrations. Counterintuitively, however, *β*3-AR agonists suppress pathological retinal angiogenesis (17, 31), whereas *β*3-AR antagonists exert mild pro-angiogenic offsetting effects. Under hypoxic OIR conditions, retinal *β*3-AR mRNA expression is significantly upregulated (31), yet the pro-angiogenic signal mediated by overexpressed *β*2-AR dominates pathological vascular proliferation (32), so the net anti-neovascularization effect of non-selective propranolol (which blocks all three *β*-subtypes) remains intact even though it also blocks the anti-angiogenic *β*3-AR signaling.

### 4.2 Core multi-target signaling cascades inhibited by beta-blockers

#### 4.2.1 Pathway description

Under hypoxic microenvironment, endogenous catecholamine agonists bind *β*1/β2/*β*3-AR on retinal Müller cells and endothelial cells to initiate multiple pro-damage cascades: (1) Gs-coupled *β*1/β2-AR activates AC-cAMP-PKA signaling, upregulating transcription factors CREB and HIF-1*α* to drive massive VEGF gene expression (33, 34); (2) receptor signaling stimulates NADPH oxidase to generate excessive ROS (35), inducing oxidative stress and retinal inflammation (36); (3) eNOS overactivation produces pathological levels of NO, worsening vasodilation and vascular leakage (37). These hypoxia-initiated signaling cascades are depicted in Figure 1 and are primarily derived from OIR animal model findings. Notably, direct confirmation of these mechanisms in human preterm infant retinas is still lacking, which constitutes an important limitation.

Beta-blockers act as competitive antagonists to occupy *β*-AR binding sites, suppressing all upstream pro-angiogenic signals simultaneously: reducing intracellular cAMP and PKA activity, inhibiting PI3K/Akt/ERK cascades (38), destabilizing HIF-1*α* (39), and lowering ROS and NO production. Meanwhile, beta-blockers upregulate tight junction protein Occludin to restore BRB integrity and reduce albumin leakage (39). Untreated ROP exhibits severe vascular leakage and disorganized pathological neovascularization (40); beta-blocker intervention restores physiological retinal vascular homeostasis, alleviates inflammatory injury, and improves electroretinogram (ERG) a- and b-wave amplitudes to realize retinal neuron protection (39, 40). All therapeutic signaling pathways shown in Figure 1 are based on OIR animal experiments; the retinal neuronal protection indicated by ERG improvements has not been directly verified in human preterm infants.

**Figure 1:**
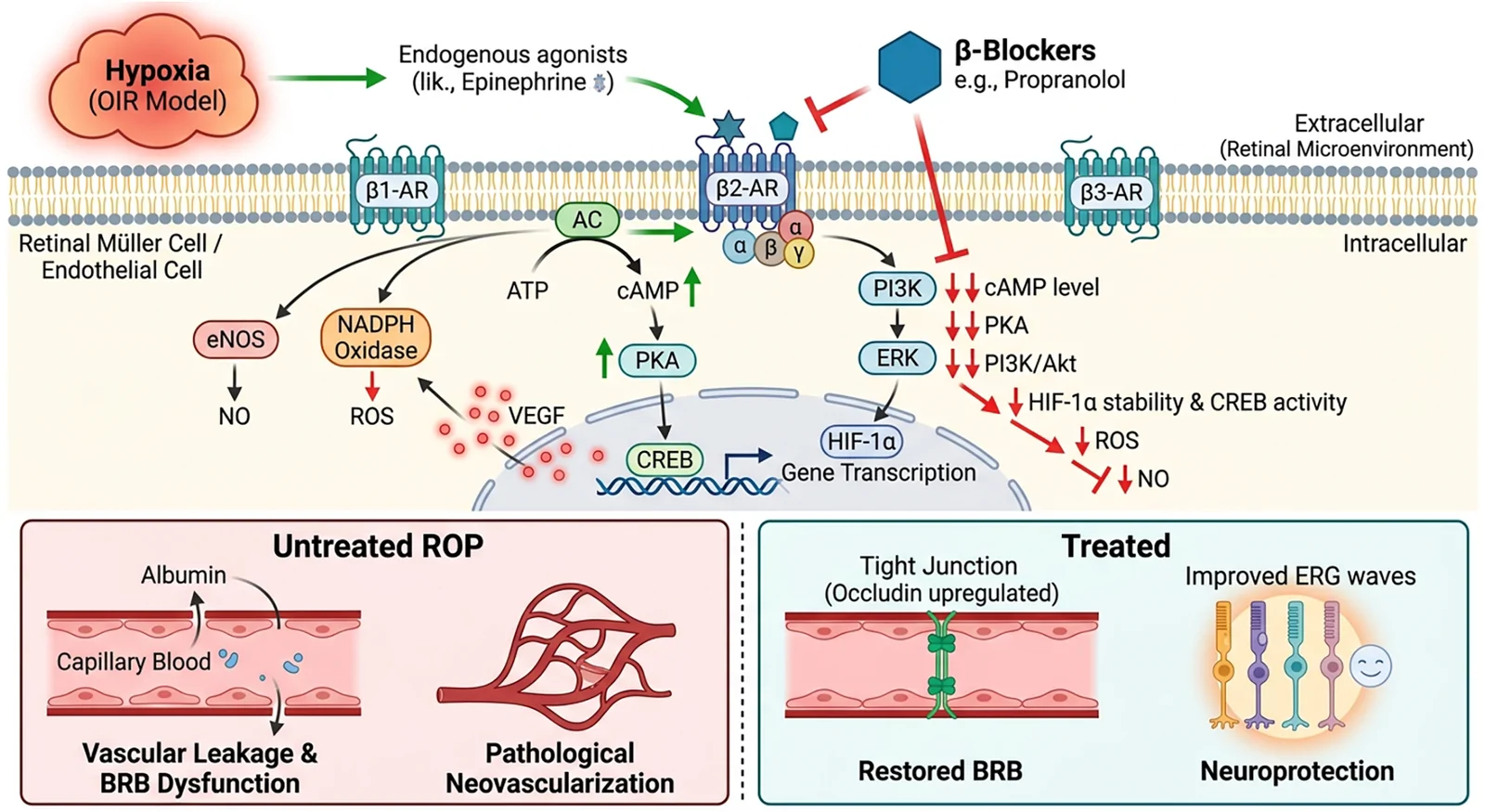
Preclinical mechanistic model of beta-blocker action in ROP derived from OIR animal experiments. Abbreviation definitions: AR, adrenergic receptor; AC, adenylyl cyclase; cAMP, cyclic adenosine monophosphate; PKA, protein kinase A; CREB, cAMP response element-binding protein; HIF-1*α*, hypoxia-inducible factor-1*α*; VEGF, vascular endothelial growth factor; PI3K, phosphatidylinositol 3-kinase; Akt, protein kinase B; ERK, extracellular signal-regulated kinase; NADPH oxidase, nicotinamide adenine dinucleotide phosphate oxidase; ROS, reactive oxygen species; eNOS, endothelial nitric oxide synthase; NO, nitric oxide; BRB, blood-retinal barrier; ERG, electroretinogram; OIR, oxygen-induced retinopathy.

Specifically, beta-blockers interrupt hypoxia-driven pathological angiogenesis via four synergistic downstream pathways: (1) Inhibition of HIF-1*α*/VEGF axis: By suppressing PI3K/Akt/ERK signaling, beta-blockers reduce HIF-1*α* protein stability and transcriptional activity, cutting off VEGF overexpression at the transcriptional level, the core driver of ROP lesions (preclinical evidence from OIR models) (40, 41). (2) Anti-oxidative and anti-inflammatory effects: Beta-blockers inhibit NADPH oxidase activity to reduce ROS accumulation, suppress microglial activation and downstream pro-inflammatory cytokine release, mitigating high-oxygen induced retinal oxidative inflammatory injury. (3) Blood-retinal barrier restoration: Hypoxia downregulates Occludin tight junction proteins to trigger vascular leakage. OIR mouse experiments confirm 20 mg/kg propranolol restores Occludin expression to 77% of normal control levels, significantly reducing plasma albumin extravasation and stabilizing endothelial intercellular junctions(the restoration data are derived from OIR mouse experiments) (42). (4) Retinal neuroprotective function: Selective *β*2-AR blockade improves ERG a- and b-wave amplitudes, protecting photoreceptors and bipolar neurons independently of anti-angiogenic effects. It should be noted, however, that Figure 1 only indicates ERG improvement at a general level without specifying these cellular targets, and this finding is based on OIR animal experiments (Figure 1) (38, 39).

## 5 Comparative efficacy of oral and topical ophthalmic medications

### 5.1 Efficacy of oral administration

Oral beta-blockers, particularly propranolol, are long-established cardiovascular medications; following the discovery of their significant efficacy in treating IH, their anti-angiogenic effects have attracted considerable attention. If proven safe and effective in ROP, this systemically administered therapy could offer a non-invasive and easily implementable treatment or preventive strategy.

Oral beta-blockers, particularly propranolol, primarily act by blocking *β*-adrenergic receptors, especially *β*2-AR, thereby effectively downregulating VEGF expression. They may also synergistically inhibit downstream signaling pathways such as PI3K/Akt to suppress abnormal retinal neovascularization. While these mechanisms have been largely validated in OIR animal models, their precise effects in the retinas of human preterm infants remain to be further elucidated (39, 40). In recent years, numerous randomized controlled trials (RCTs) have been conducted worldwide to evaluate the efficacy of oral propranolol in the prevention or treatment of ROP, but inconsistent clinical outcomes and uneven research quality are prominent problems in existing evidence. Makhoul JR (43) et al. conducted an exploratory pilot RCT (Table 1) with a dosage regimen of 2.0 mg/kg/day. The study found that the rate of ROP progression in the propranolol group (30%) was significantly lower than that in the placebo group (66%), this suggests that oral propranolol may have some efficacy in preventing the progression of ROP. This pilot study carries methodological limitations including small sample size and single-center design, with a heightened risk of selection and measurement bias. A randomized clinical trial conducted in Iran evaluating the efficacy of oral propranolol in the treatment of existing ROP found that 81.34% of infants with ROP in the treatment group recovered, compared with 66.7% in the control group; however, this difference was not statistically significant (*P* > 0.05). In this small-sample study, the therapeutic advantage of propranolol was not statistically confirmed (44). The contradictory results observed between these two studies can be explained by multiple confounding factors. Firstly, the Iranian study enrolled a smaller sample size, resulting in insufficient statistical power to detect inter-group differences. Secondly, there were differences in gestational age, birth weight, and baseline ROP staging of the included preterm infants across the studies. Thirdly, inconsistent treatment cycles and follow-up time points may also have interfered with the stability of clinical outcomes. Collectively, such small-sample, single-centre studies are susceptible to type II statistical errors, and their credibility is low. A 2021 meta-analysis that included five eligible RCTs found that, compared with the control group, oral propranolol significantly reduced the risk of ROP progression; furthermore, the proportion of infants in the propranolol treatment group requiring follow-up treatment (such as laser photocoagulation or anti-VEGF injections) was significantly lower than that in the control group (45). A systematic review and meta-analysis designed to evaluate the preventive efficacy of oral propranolol similarly concluded that this agent may significantly prevent severe ROP (46). A systematic review and meta-analysis sought to investigate the effects of different doses of propranolol on treatment outcomes, incorporating a total of 14 studies (10 of which were RCTs) involving 474 patients. The analysis found that for more advanced stages of ROP (such as Stage 2 and Stage 3), higher doses of oral propranolol (e.g., 2 mg/kg/day) may be more effective than lower doses (47). Nevertheless, these meta-analyses exhibit unavoidable methodological defects, thereby restricting the overall evidence strength. The majority of relevant original investigations are small-scale preliminary trials. Noticeable variations exist in study design, participants' baseline characteristics, and medication administration protocols across individual works. Moreover, substantial clinical heterogeneity can be observed among these studies. Existing small- and medium-sized RCTs and several meta-analyses suggest that oral propranolol may effectively reduce the risk of ROP progression and decrease the need for invasive treatment in affected infants. Many published analyses lack robust subgroup and sensitivity analyses to account for potential confounders. Taken together, the certainty of evidence supporting relevant conclusions is judged as low to moderate according to the GRADE (Grading of Recommendations Assessment, Development and Evaluation) framework. However, this 2021 meta-analysis clearly states that while propranolol is effective, it also significantly increases the risk of adverse events such as bradycardia, hypotension, bronchospasm, apnea, and hypoglycemia (45). The safety risk is particularly prominent in preterm populations due to their immature physiological function. Propranolol undergoes extensive first-pass metabolism primarily in the liver via the cytochrome P450 enzyme system (mainly CYP2D6 and CYP1A2) (48), however, the hepatic enzyme system in preterm infants is not yet mature, and the activity of enzymes such as CYP2D6 is very low at birth, taking several months or even longer to develop to adult levels. The risks of foreseeable systemic adverse reactions, such as bradycardia, hypotension, hypoglycemia, and bronchospasm, are particularly pronounced in the vulnerable population of preterm infants and may even lead to fatal outcomes (45).

**Table 1:** Summary of major clinical studies of beta-blockers for ROP.

Study/trial	Study design	Sample size	Gestational age	ROP stage	Route	Dose/duration	Primary outcome	Adverse events	Limitations
Makhoul JR et al.	Exploratory pilot RCT	Small (exact number not specified)	Not specified	Not specified (ROP progression assessed)	Oral propranolol	2.0 mg/kg/day	ROP progression rate: 30% in propranolol group vs. 66% in placebo group (significant)	Not described in detail	Small sample, single-center, lack of strict sample size calculation, no long-term follow-up, high risk of selection and measurement bias
Iranian RCT	Randomized clinical trial	Small	Not specified	Established ROP (specific stages not stated)	Oral propranolol	Not specified	Recovery rate: 81.34% in treatment group vs. 66.7% in control group; not statistically significant (*P* > 0.05)	Not described in detail	Small sample with insufficient statistical power, differences in gestational age/birth weight/baseline ROP stage across studies, inconsistent treatment cycles and follow-up time points
2021 Meta-analysis	Meta-analysis (5 RCTs)	5 RCTs	Not specified	Not specified (ROP progression assessed)	Oral propranolol	Not specified	Significantly reduced risk of ROP progression; significantly lower proportion of infants requiring subsequent treatment (e.g., laser photocoagulation or anti-VEGF injections)	Bradycardia, hypotension, bronchospasm, apnea, hypoglycemia	Most original studies were small-scale preliminary trials; notable variations in study design, baseline characteristics, and medication protocols; substantial clinical heterogeneity; lack of subgroup and sensitivity analyses
Dose-related Meta-analysis	Meta-analysis (14 studies, 10 RCTs)	474 patients	Not specified	Includes Stage 2 and Stage 3	Oral propranolol	Comparison of different doses (e.g., 2 mg/kg/day vs. lower doses)	Higher doses may be more effective than lower doses for more advanced ROP (Stage 2, 3)	Not described in detail	Uneven research quality, notable clinical heterogeneity, limited number of high-quality RCTs
ROPROP Phase III trial	International, multicenter, randomized, double-blind, placebo-controlled	Not specified	<28 weeks (extremely preterm)	Not specified (progression to treatment threshold Grade 3 assessed)	Oral propranolol	Not specified	Final results not yet formally published	Not specified	Results unpublished; unable to assess
0.1% propranolol eye drops trial	Early exploratory clinical trial	Not specified	Preterm infants	Early-stage ROP	Topical eye drops	0.1% concentration	Extremely well tolerated; insufficient efficacy in inhibiting ROP progression; failed to reduce the rate of disease progression	Extremely well tolerated	Small sample, insufficient efficacy, no long-term data
0.2% propranolol eye drops Phase II trial	Multicenter Phase II clinical trial	Larger than 0.1% trial (exact number not specified)	Preterm infants	Stage 1	Topical eye drops	0.2% concentration	Significantly reduced risk of progression to Stage 2 or 3 (risk ratio 0.521, 95%CI: 0.297–0.916)	No serious cardiovascular adverse events; plasma concentrations well below 20 ng/mL safety threshold	Phase II preliminary research, no long-term follow-up data, no long-term visual and neurodevelopmental outcome data
Network meta-analysis (0.2% eye drops vs. oral)	Network meta-analysis	Not specified	Not specified	Stage 2–3 (advanced ROP)	0.2% eye drops vs. oral 1.5 mg/kg/day	Not specified	Comparable efficacy between 0.2% eye drops and 1.5 mg/kg/day oral (OR=0.37 vs. 0.14)	Significantly lower risk of systemic adverse reactions with eye drops	Limited number of high-quality RCTs, obvious clinical heterogeneity, conclusions with certain contingency requiring further verification

Currently, there is no consensus regarding the optimal dosage, timing of treatment, or appropriate patient population for propranolol, and much of the evidence comes from small pilot studies with methodological limitations (39). An international, multicenter, randomized, double-blind, placebo-controlled Phase III ROPROP clinical trial (39, 49), designed to evaluate the safety and efficacy of oral propranolol in preventing the progression of ROP to the Stage 3 requiring treatment in extremely preterm infants (gestational age < 28 weeks), has not yet had its final results formally published in a peer-reviewed journal. The results of this trial are expected to provide the strongest evidence to date regarding the role of oral propranolol in the treatment of ROP, potentially revolutionizing or even ending the clinical application of this therapy. The clinical evidence discussed in this section primarily falls under the category of preventing the progression of established ROP; evidence for primary prevention in cases where ROP has not yet developed remains lacking.

In summary, existing preliminary evidence suggests that oral propranolol can inhibit ROP pathological angiogenesis and reduce disease progression and invasive treatment rate, but the overall evidence strength is low, the research quality is uneven, and there are widespread biases and unresolved clinical controversies. Combined with the unclear long-term visual and neurodevelopmental prognosis data and unstandardized medication scheme, oral propranolol can only be defined as an experimental research therapy at present, and cannot be used as a standard conventional treatment for ROP.

### 5.2 Efficacy of topical ophthalmic medications

Systemic adverse reactions associated with oral propranolol regimens are a significant concern that cannot be overlooked (50). Several clinical trials and meta-analyses evaluating the efficacy of oral propranolol for the treatment of ROP have reported inconsistent results, but safety concerns remain a persistent issue. Due to its significant cardiovascular and respiratory side effects, oral administration is limited in the neonatal population; researchers have proposed the concept of topical administration (17). Topical ocular administration, as an innovative delivery method capable of delivering drugs directly to the target organ (the eye/retina) while minimizing systemic exposure and thereby reducing the adverse effects associated with oral medication, has gradually become a focus of research. In a rat OIR model, topical administration (0.2% propranolol ophthalmic solution) improves retinal vascular damage and preserves astrocyte templating by reducing VEGF expression and upregulating soluble VEGFR-1 (51). The success of local drug delivery depends on whether the drug can penetrate barriers such as the cornea and the anterior chamber to reach the retina in the posterior segment. Studies typically use microdroplet technology to ensure precise dosing, with concentrations primarily ranging from 0.1% to 0.2%. However, the translational efficiency of preclinical animal research to clinical practice is limited. The corneal barrier, anterior chamber barrier and vitreous barrier of preterm infants are different from adult animals, resulting in uncertain ocular penetration efficiency of topical drugs, which is an important inherent limitation of preclinical research.

Current clinical studies on topical propranolol have significant dose-efficacy differences and research quality defects. An early exploratory clinical trial evaluated the efficacy of 0.1% propranolol eye drops in preventing the progression of early-stage ROP. The results indicated that the 0.1% concentration was extremely well tolerated in preterm infants; however, its efficacy in inhibiting the progression of ROP was insufficient, and it failed to reduce the rate of disease progression (52). Subsequent researchers conducted a multicenter Phase II clinical trial evaluating the safety and efficacy of 0.2% propranolol eye drops in preventing the progression of Stage 1 ROP to Stage 2 or 3 in preterm infants. The results showed that treatment with 0.2% propranolol eye drops significantly reduced the risk of disease progression to Stage 2 or 3 (risk ratio 0.521, 95%CI: 0.297–0.916). No serious cardiovascular adverse events were observed, and plasma concentrations remained well below the safety threshold of 20 ng/mL (53). Compared with early exploratory trials, this study has a larger sample size and multicenter design, which reduces single-center bias and improves evidence reliability, but it still belongs to phase II preliminary research without long-term follow-up data. Almost none of the existing studies provide systematic data on long-term (≥12 months) visual function (such as corrected visual acuity, refractive status, and visual field) in children following topical propranolol treatment. Currently, dose optimization remains a key challenge in the development of ophthalmic formulations. The 0.1% eye drops have been found to be ineffective and are therefore not suitable for clinical use (52). A network meta-analysis showed that in the late stages of ROP (S2-3), the efficacy of 0.2% eye drops was comparable to that of 1.5 mg/kg/day oral medication (OR=0.37 vs. 0.14), and the risk of systemic adverse reactions was significantly reduced (47). This result clarifies the dose advantage and safety superiority of topical drugs, but the meta-analysis is limited by the small number of included high-quality RCTs, with obvious clinical heterogeneity among studies, and the conclusion has certain contingency and needs further verification. The late stage of ROP (stages S2-3) represents the optimal window for intervention with oral and topical propranolol. At this stage, *β*2-AR expression is upregulated and VEGF is highly expressed; the drug exerts its effects by inhibiting vascular endothelial cell migration and lumen formation. However, the efficacy of the drug is limited in the early stages (stages S0-1), which may be related to the fact that abnormal blood vessels have not yet begun to proliferate (47, 54). To date, the vast majority of studies on topical eye drops for ROP have been limited to propranolol at concentrations of ≤0.2%; there are no published clinical studies on the use of higher concentrations of propranolol eye drops for ROP. Although topical propranolol offers safety advantages, its long-term efficacy and optimal dosing regimens (such as frequency and duration of treatment) still require validation. The clinical evidence discussed in this section primarily falls under the category of preventing the progression of established ROP; evidence for primary prevention in cases where ROP has not yet developed remains lacking.

In conclusion, topical propranolol has outstanding safety advantages over oral administration and definite short-term efficacy in advanced ROP, but the existing research has problems such as uneven quality, widespread bias, insufficient sample size, lack of standardized medication schemes and blank long-term prognosis data. Existing evidence discrepancies largely stem from heterogeneous study designs, inconsistent drug concentrations and treatment durations, and differing baseline characteristics of enrolled infant (49, 53).

## 6 Summary

Abundant OIR animal experiments provide consistent preclinical evidence supporting the anti-angiogenic, barrier-protective, anti-inflammatory and neuroprotective activities of beta-blockers. The molecular mechanism relies on blocking *β*-adrenergic signaling to suppress the HIF-1*α*/VEGF hypoxia-driven pathological angiogenesis axis, forming solid preclinical support for ROP intervention. Functional divergence of *β*1/β2/*β*3 receptor subtypes explains partial inconsistent mechanistic observations, with *β*2-AR confirmed as the dominant therapeutic target.

Despite over a decade of translational research, beta-blockers have not yet achieved routine clinical adoption for ROP. Existing clinical evidence remains preliminary, and the following barriers persist: (1) efficacy has not been consistently demonstrated across trials, with oral propranolol responses varying by gestational age, ROP stage, and dose (39), while topical preparations show concentration-dependent effects—0.1% is ineffective, and only 0.2% has shown preliminary, short-term benefits for Stage 2–3 lesions in a single Phase II trial (53); (2) unresolved safety concerns, including life-threatening systemic adverse events from oral propranolol and an absence of long-term visual and neurodevelopmental data for topical formulations; (3) no consensus exists on treatment timing, dosing, duration, or eligible patient subgroups, and Phase III evidence remains insufficient; (4) strong competition from established therapies—laser photocoagulation and intravitreal anti-VEGF injection—which, despite being the current standard of care, carry their own unresolved safety considerations: laser causes permanent retinal tissue damage and cannot treat the peripheral avascular retina without ablation; intravitreal anti-VEGF injection is invasive, with risks of endophthalmitis, potential systemic VEGF suppression of uncertain long-term consequence, and the need for repeated anesthesia in preterm infants. When interpreting these barriers, three distinct clinical indications must be distinguished. For primary prevention (preventing the onset of ROP in at-risk preterm infants), no clinical trial has specifically evaluated this indication, and no conclusion regarding primary preventive value should be drawn. For prevention of progression(halting the advancement of existing ROP), the category for which most current clinical evidence demonstrates preliminary efficacy with low-to-moderate certainty, the GRADE framework was used in a previously published systematic review (45) to rate the efficacy of oral propranolol for preventing ROP progression. For treatment of established ROP, the only directly relevant trial failed to confirm therapeutic efficacy, and a network meta-analysis suggesting comparable efficacy between topical and oral formulations is constrained by high clinical heterogeneity. Under current evidence, beta-blockers cannot be regarded as conventional treatment for ROP and should be positioned only as experimental early intervention candidates.

Major evidence gaps remain. The most critical is the absence of large-cohort longitudinal data on visual acuity, refractive development, and neurocognitive outcomes in beta-blocker-treated preterm infants. Key future priorities include: publishing the multicenter Phase III ROPROP trial; launching multicenter double-blind Phase III trials of 0.2% propranolol eye drops; developing retina-targeted formulations to improve posterior segment penetration; and establishing longitudinal cohorts with at least 3–5 years of follow-up. Addressing these gaps is essential before beta-blockers can be positioned as a viable alternative to current first-line ROP therapies.

A balanced comparison of the four therapeutic modalities reveals distinct safety profiles and evidence gaps. Oral propranolol carries well-documented risks of bradycardia, hypotension, bronchospasm, apnea, and hypoglycemia—particularly hazardous in preterm infants with immature hepatic enzyme systems (as detailed in Section 5.1)—yet lacks long-term visual and neurodevelopmental outcome data. Topical ocular beta-blockers minimize systemic exposure and have shown no serious cardiovascular adverse events in a Phase II trial, with plasma concentrations well below the safety threshold of 20 ng/mL; however, long-term follow-up data remain absent. Laser photocoagulation, while a standard therapy with decades of use, causes permanent retinal tissue destruction and provides no benefit to the avascular peripheral retina other than ablation. Intravitreal anti-VEGF injection effectively controls neovascularization but carries procedural risks of endophthalmitis, requires repeated anesthesia, and raises unresolved questions regarding systemic VEGF suppression and its potential impact on developing organs. Each modality thus presents distinct risk-benefit trade-offs, and the evidence base for long-term safety is incomplete for all four approaches, albeit to varying degrees.

Beta-blockers should be positioned as experimental early intervention candidates rather than as established preventive, adjunctive, or alternative therapies for ROP. Evidence for treatment of established ROP is inconclusive, and beta-blockers cannot replace laser photocoagulation or intravitreal anti-VEGF injection as standard care. Their potential role should be investigated within rigorously designed clinical trials until sufficient evidence from large-scale, multicenter Phase III studies with long-term follow-up becomes available.

Most of the clinical evidence included in this review comes from Asian, European, and American populations; currently, there is virtually no research on beta-blockers in relation to ROP in preterm infants in Africa, and there is a significant gap in evidence regarding racial and regional differences. Existing studies on retinopathy of prematurity (ROP) in African preterm infants have primarily focused on epidemiological screening. A systematic review and meta-analysis of sub-Saharan Africa evaluated the prevalence and risk factors of ROP in the region (55); Epidemiological studies have also clearly identified Africa as an “emerging hotspot” for preventable blindness caused by ROP?. In terms of prevalence, there are significant differences among African countries: some studies have reported a prevalence of ROP as high as 38.1% (56); a study conducted at a tertiary hospital in South Africa also assessed the prevalence, risk factors, and treatment outcomes of ROP in that region (57). Given that the genetic background, birth weight distribution, and care resources of the African preterm infant population differ significantly from those in Asia, Europe, and the United States, there is a severe lack of evidence regarding the efficacy and safety of beta-blockers for the treatment of ROP in this population. Therefore, extreme caution should be exercised when extrapolating the conclusions of this review to the African population, and there is an urgent need for future studies on pharmacological treatment of ROP in African preterm infants.

## Method of literature search

This narrative review was based on a comprehensive literature search of PubMed, Web of Science, and Embase databases, spanning all available publications from database inception to March 2026. The search strategy integrated relevant Medical Subject Headings (MeSH) terms and free keywords, including retinopathy of prematurity (ROP), beta-blockers, propranolol, *β*-adrenoceptor, and oxygen-induced retinopathy. Only English-language publications were considered for inclusion. To achieve a holistic understanding of the current research status in this field, we did not apply strict limitations on study design. The included literature encompassed diverse research forms, such as clinical trials, observational studies, animal and mechanistic studies, systematic reviews and meta-analyses, as well as case reports. Furthermore, the reference lists of eligible articles were manually reviewed to identify additional eligible studies and consolidate the evidence base of this review.

GRADE Evidence Assessment: This narrative review did not perform an independent Grading of Recommendations Assessment, Development and Evaluation (GRADE) assessment of the included studies. References to “low to moderate certainty of evidence” or similar GRADE-based descriptors throughout this review are cited from previously published systematic reviews and meta-analyses that formally conducted GRADE evaluations, and the corresponding source references should be consulted for the original assessment methodology and detailed evidence profiles.
